# Is there a role for rechallenge and reintroduction of anti-EGFR plus chemotherapy in later lines of therapy for metastatic colorectal carcinoma? A retrospective analysis

**DOI:** 10.3332/ecancer.2020.1069

**Published:** 2020-07-13

**Authors:** Amanda Karani, Tiago Cordeiro Felismino, Lara Diniz, Mariana Petaccia Macedo, Virgilio Souza e Silva, Celso Abdon Mello

**Affiliations:** 1 Department of Medical Oncology, AC Camargo Cancer Center, Sao Paulo 01509-000, Brazil; 2 Department of Pathology, AC Camargo Cancer Center, Sao Paulo 01509-000, Brazil; a https://orcid.org/0000-0002-8315-1562

**Keywords:** colorectal carcinoma, treatment, prognosis, anti-EGFR, rechallenge

## Abstract

**Background:**

Mechanisms of resistance have been described during disease progression (PD) for patients under treatment with anti-EGFR plus chemotherapy (CT). The aim of our study was to evaluate efficacy of anti-EGFR rechallenge (ReCH) and reintroduction (ReIn) in metastatic colorectal cancer (mCRC).

**Materials and methods:**

This is a retrospective analysis of patients with mCRC that previously received anti-EGFR + CT and interrupted therapy due to PD in the ReCH group and other reasons in the ReIn group. We aimed to describe progression-free survival (PFS), overall survival (OS) and response rate (RR) after re-exposure and to evaluate prognostic factors associated with PFS.

**Results:**

Sixty-eight patients met the inclusion criteria. The median follow-up after re-exposure was 39.3 months. ReCH was adopted in 25% and ReIn in 75%. The median anti-EGFR free interval was at 10.5 months. At re-exposure, the main CT regimen was FOLFIRI in 58.8%. Cetuximab and Panitumumab were used in 59 and 9 patients, respectively. mPFS for ReCH and ReIn was 3.3 × 8.4 months, respectively (*p* 0.001). The objective response rate for ReCH and ReIn was 18% and 52%, respectively. In univariate analysis, adverse prognostic factors related to PFS were: stable disease or PD at first anti-EGFR exposure (HR: 2.12, CI:1.20–3.74; *p* = 0.009); ReCH (HR: 3.44, CI:1.88–6.29, *p* < 0.0001); rechallenge at fourth or later lines (HR: 2.51, CI:1.49–4.23, *p* = 0.001); panitumumab use (HR: 2.26 CI:1.18–5.54, *p* = 0.017). In the multivariate model, only ReCH remained statistically significant (HR = 2.63, CI: 1.14–6.03, *p* = 0.022).

**Conclusion:**

In our analysis, ReCH resulted in short PFS and low RR. However, reintroduction of anti-EGFR plus CT before complete resistance arose resulted in prolonged PFS. These data could be clinically useful to guide a treatment break due to side effects or patient decisions. Our data should be confirmed by larger and prospective trials.

## Introduction

Colorectal cancer is a leading cause of cancer-related death [1]. Approximately 25% of all colon cancer patients will have advanced disease at diagnosis and almost 50% will eventually recur during the course of their disease [2]. Progress in systemic treatment seen in recent years has resulted in prolonged survival for patients with metastatic disease [2].

In the metastatic setting, EGFR pathway plays an important role driving cancer cell growth and survival [3]. Approximately 60% of metastatic colorectal cancer (mCRC) tumors develop mutations in the EGFR pathway (mainly KRAS/NRAS/BRAF) which will lead to a constitutively downstream activation and primary resistance to anti-EGFR agents [4]. On the other hand, the remaining 40% of all mCRC patients will derive benefit from these agents. Large phase 3 trials demonstrated that first-line anti-EGFR agents (cetuximab and panitumumab) plus standard chemotherapy (FOLFOX and FOLFIRI) induced objective responses in about 60% of patients. The median progression-free survival (PFS) was 10 months and the median overall survival (OS) surpassed 30 months [5–8].

However, two main issues will inexorably emerge with anti-EGFR agents: toxicity and acquired resistance. For instance, cutaneous toxicity might be an early side effect that significantly impacts quality of life and results in drug discontinuation [9]. Moreover, a series of acquired resistance mechanisms have been recently proposed that lead to progressive disease and consequently the need of alternative drugs [10].

In real-world care of patients with mCRC, toxicity and resistance are the main reasons for anti-EGFR discontinuation. The resection of all macroscopic diseases is also a common cause of suspension of these agents as clinical trials show a lack of benefit of these drugs in the “adjuvant” setting [11]. It could be argued that after an anti-EGFR free interval, toxicity is mitigated and resistant clones are reduced [12]. As there is still a paucity of effective treatment lines, re-challenging and reintroduction of previous drugs may become an important strategy (cancer treat review). We have previous shown that re-challenging patients with oxaliplatin containing regimen resulted in prolonged survival for a selected and heavily treated group of patients [13]. However, anti-EGFR rechallenge is currently under investigation. The aim of this study is to retrospectively evaluate the efficacy of anti-EGFR rechallenge and reintroduction after a previous exposure in real-word metastatic colon cancer patients.

## Patients and methods

This is a single-centre, retrospective analysis that aimed to evaluate the efficacy of anti-EGFR rechallenge and reintroduction in real-world patients with metastatic colorectal cancer. The study was approved by the local ethics committee.

Inclusion criteria were: patients with histologically confirmed metastatic colorectal adenocarcinoma, KRAS or KRAS/NRAS wild-type (BRAF status was not mandatory), first treatment with anti-EGFR agent (cetuximab or panitumumab) plus chemotherapy for at least 3 months, re-exposure therapy after a minimum of 3 months since the last dose of anti-EGFR (a stop period), re-exposure to anti-EGFR plus chemotherapy duration for at least 2 months. Patients were grouped according to reason to discontinuation: group 1 due to progression disease or rechallenge (ReCH) and group 2 due to other reasons (toxicity, medical doctor preference or treatment holiday, metastasectomy) or reintroduction (ReIn)

Clinical data were retrospectively collected from medical records. Our primary endpoint was PFS, defined as the time from anti-EGFR re-exposure start until disease progression or death from any cause. Secondary endpoints were OS after anti-EGFR re-exposure, response rate by RECIST criteria [14] at the anti-EGFR re-exposure. We also aimed to evaluate clinical, pathological and treatment variables as prognostic factors during the anti-EGFR re-treatment.

### Statistics

Survival curves were estimated using the Kaplan–Meier method and compared with log-rank test. Univariate and multivariate prognostic analysis for PFS were performed using the Cox regression method. Variables included in our model were: reason for first discontinuation (progressive disease × other reason); objective response at first anti-EGFR exposure (defined as complete response plus partial response); line of anti-EGFR re-exposure (second + third × fourth later); antibody (cetuximab × panitubumab); metastasis sites (liver only x lung only × other) and anti-EGFR free interval (<6m × >6m). All variables considered statistically significant in the univariate analysis were included in the multivariate analysis. All tests were considered statistically significant with a two-sided *p*-value of <0.05. Statistical analysis was performed with IBM SPSS 20.

## Results

From 2009 to 2017, we identified 68 patients who met our inclusion criteria. Clinical and pathologic characteristics are shown in Table 1. The median follow-up time from re-exposure was 39.3 months (95% CI: 26.7–51.9). The mean age was 56 years (29–85). Left-sided primary was found in 64 patients. Rechallenge was performed in 17 (25%); in the ReIn group, reasons for discontinuation anti-EGFR during first exposure was chemotherapy holiday in 23 (33.8%), metastasectomy in 12 (17.6%) and toxicity in 6 (8.8%). Anti-angiogenic therapy prior to re-exposure was employed in 40%. Median anti-EGFR free interval was 10.5 months (95% CI: 8.8–12.1) as shown in Table 2. Metastasectomy was frequent in this cohort of patients (61%). Liver, lung, peritoneum, combined liver + lung + other sites metastasectomies were performed in 76.1%, 11.9%, 4.7%, 7.1%, respectively (Table 1). As seen in Table 1, more patients were submitted to metastasectomy in the ReIn group as compared to Re-Ch (35 × 7 patients, respectively). Post re-exposure metastasectomy was not frequent and only 1 patient in the ReCH group and 2 patients in the ReIn group were submitted to liver metastasectomy after re-exposure. We found a relatively high number of second metastastasectomy in the ReIn as compared to the ReCH group (16 × 2 patients, respectively). However, only 1 patient in the ReIn group had second metastasectomy after re-exposure. All RAS status was known in 46% in the ReIn group and only KRAS wild type was seen in 3% in the ReCH group. During first treatment with anti-EGFR plus chemotherapy, 66.6% of patients in the ReIn group achieved PR as compared to only 31.3% of patients in the ReCH group.

At re-exposure, cetuximab and panitumumab were used in 86.8% and 13.2% of cases, respectively. No patient in the ReCH group used panitumumab in the re-exposure (Table 2). The main chemotherapy backbone was FOLFIRI in 58.8%. The median re-exposure line was the fourth. However, 72.6% of patients in the ReIn group were re-exposed in the second and third line and 70.6% in ReCH received in the fourth line.

**Figure 1.**
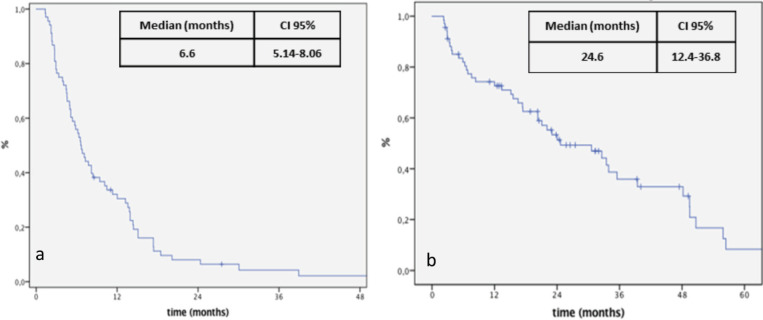
Kaplan-Meier curve for PFS (a) and OS (b) after re-exposure to anti-EGFR + chemotherapy.

**Figure 2.**
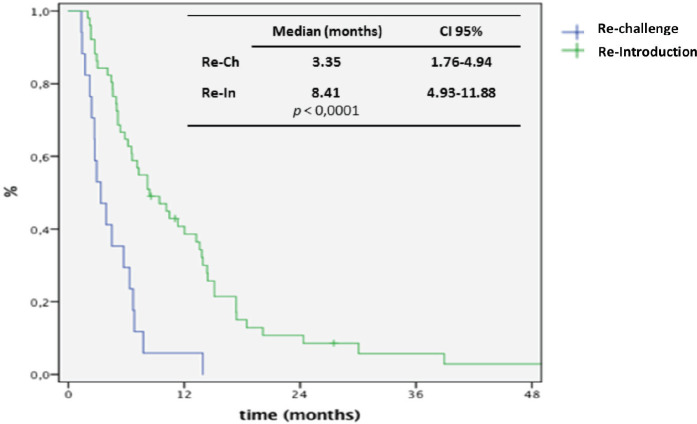
Kaplan-Meier curve for PFS for ReCH and ReIn.

At the time of data analysis, 64 events occurred. The median OS since the first line for the entire group was 70 months. After re-exposure, the median PFS was 6.6 months (95% CI: 5.1–8.0) and the median OS was 24.4 months (95% CI: 12.4–36.8) (Figure 1). The median PFS was 3.3 and 8.4 months for ReCH and ReIn, respectively (*p* < 0.001), as shown in Figure 2, and the median OS was 7.5 and 33.4 months for ReCH and ReIn, respectively (*p* = 0.005). For the ReIn group, median PFS for CR/PR × SD during first exposure was 8.4 × 4,9 months, respectively (*p* = 0.083), previous bevacizumab versus no bevacizumabe was 6.1 × 10.4 months, respectively (*p =* 0.082), interval for reintroduction <6 months × >6 months was 7.2 × 8.4 months (*p* = 0.083). For the ReCH group, no significant difference was seen in PFS according to these variables (response to previous anti-EGFR (*p* = 0.06), previous bevacizumab (*p* = 0.07) and interval for reintroduction < 6 months (*p* = 0.16). Response evaluations (Table 1) were available in 67 cases and were as follows: complete response 3%, partial response 40.3%, SD 41.8% and progressive disease 14.9%.

We further performed a second analysis excluding three patients who underwent metastasectomy (two in the ReIn group and one in ReCH group), and the mOS was 6.86 months (95% CI: 4.77–8.96) for rechallenge versus 33.47 (95% CI: 23.08–43.86) for reintroduction (*p* = 0.004) and mPF was 2.92 (95% CI: 1.70–4.14) for rechallenge versus 8.18 (95% CI: 5.02–11.33) for reintroduction (*p* < 0.0001).

### Univariate analysis for PFS

In our univariate model (shown in Table 3), main prognostic factors were: progressive disease as reason for first anti-EGFR discontinuation (HR: 3.44, 95% CI 1.88–6.29, *p <* 0.0001); rechallenge at the fourth line or later lines (HR: 2.51, 95% CI 1.49–4.23, *p =* 0.001); panitumumab use (HR: 2.56, 95% CI 1.18–5.54, *p =* 0.017) and absence of clinical benefit at first anti-EGFR exposure (HR: 2.12, 95% CI 1.20–3.74, *p =* 0.009). Anti-EGFR free interval (*p =* 0.67) and sites of metastasis were not related to prognosis (*p =* 0.16).

### Multivariate analysis for PSF

All statistically significant variables in the univariate analysis were included in the multivariate model (Table 3). PD as reason for first anti-EGFR discontinuation or ReCH remained statistically significant (HR: 2.63, 95% CI 1.14–6.03, *p =* 0.022). Rechallenge at the fourth line or later lines was marginally significant (HR: 1.18, 95% CI 0.99–3.32, *p* = 0.053).

## Discussion

Rechallenge and reintroduction of previous agents is a relatively common practice in real-world oncology [15]. In the present study, we found that ReIN of anti-EGFR antibody plus chemotherapy resulted in prolonged survival. On the other hand, those patients that presented previous progression and were submitted to anti-EGFR plus chemotherapy did not seem to benefit from this strategy with a short PFS of only 3.3 months. By analysing the clinical and pathological variables of 68 re-treated patients, only the progression during previous anti-EGFR combination was associated with poor survival.

The OS of patients with mCRC has improved over the past years. The incorporation of anti-EGFR and anti-VEGF in the first and second line of treatment resulted in a median OS around 30 months [5–8; 16–20]. However, the median PFS time for the first line has been stable and is around 10 months in most of the trials. In the third line, prospective and randomised trials showed positive but modest benefit of regorafenib and tas-102 as compared to placebo [21, 22].

However, after the second and third line of therapy, a great number of patients still have a good performance status and demand for active treatment instead of best supportive care alone. As a result, re-challenging and re-introducing previous agents is a relatively common practice in the real world despite the lack of robust evidence demonstrating the efficacy of these strategies. Reintroduction is defined as re-administration of a previous agent to which tumour has not already proved to be resistant and discontinuation was not due to progression of disease (chemotherapy holliday, toxicity, metastasectomy, for example) and rechallenge is the reintroduction exposure of an agent in patients that developed resistance during prior use with progression of disease [15]. Oxaliplatin is commonly re-introducted in the third line or further lines in a group of patients that interrupted treatment due to stop-and-go strategy or due to limiting neuropathy [23]. We have previously shown that even for patients that presented disease progression during oxaliplatin-containing regimen can benefit from rechallenge [13]. The use of anti-angiogenic agents such as bevacizumab, aflibercept, ramucirumab after previous exposure and progression is supported by many prospective trials [18–20].

On the other hand, there are limited data evaluating the maintenance of anti-EGFR after disease progression [24, 25]. In this scenario, only a small phase 2 study (CAPRI—GOIM) showed small benefit in PFS in the maintenance of cetuximab associated with chemotherapy with no statistically significant benefit in OS [24]. Most of the guidelines do not recommend the maintenance of anti-EGFR inpatients with PD [2]. In the scenario of partial resistance, our data showed that patients that interrupted therapy with anti-EGFR before progression presented a prolonged PFS and OS. The response rate after reintroduction was relatively high (52% CR + PR) as compared to first- and second-line trials with the combination of anti-EGFR and chemotherapy [5, 16, 17]. It becomes more relevant if we consider that our patients were heavily pre-treated, since anti-EGFR re-exposure occurred during and after the fourth line on average. Moreover, our data could generate the hypothesis that stopping anti-EGFR is safe and could mitigate side effects and improve quality of life, since the median PFS was 8.4 months and OS was 33.4 months.

In our anaylsis, our patient population was heavily treated with systemic therapy and almost 70% had metastasectmy performed. Definitely it is responsible for a long survival time since the diagnosis of metastasis. However, it is important to note that only 3 patients (1 in the ReCH group and 2 in the ReIn group) were submitted to metastasectomy after re-exposure. Additionally, we reviewed the metastasectomy procedures and we found that the proportion of patients undergoing metastasectomy was higher in the reintroduction group. Moreover, second metastasectomy was performed in a relatively large group of patients (16 patients). It indicates that these patients have more favourable behaviour than those submitted to rechallenge. However, this finding does not invalidate our analysis, since we aim of the study is to evaluate PFS after re-exposure and after re-exposure there were insignificant number of metastasectomy and there were relevant differences in terms of OS and PFS according the re-expouse strategy (ReIn or ReCH).

Part of the failure to anti-EGFR agents is due to the emergence of acquired resistance [26–28]. Many mechanisms involved with secondary resistance have been described and most of them involve the RAS-MEK-ERK pathway [27]. The clonal selection hypothesis is suggested as the main mechanism of acquired resistance [27, 29]. It is based on tumour heterogeneity and drug induced clonal selection. Briefly, we could hypothesise that anti-EGFR sensitive clones would be controlled during cetuximab/panitumumab use. After an initial response, RASwt clones begin to be depleted and resistant clones overcome, with consequent progressive disease [26]. It has been shown that refractory patients have higher levels of mutant RAS and EGFR circulating tumour DNA [29].

Despite all of these preliminary data about anti-EGFR resistance, there is limited clinical evidence showing that the maintenance of these agents and a change in the backbone chemotherapy benefits a group of patients that experienced progression of disease. In one prospective, phase 2 study, Santini *et al* [30] evaluated 39 patients with mCRC in which rechallenge was offered to patients who developed progression during the first exposure to anti-EGFR therapy. The median re-exposure line was four, consistent with our study. The reported PFS was 6.6 months and the objective RR was 53.8% [30]. Additionally, Liu *et al* [31] conducted a retrospective analysis of 86 patients submitted to rechallenge therapy with cetuximabe and irinotecan and found a median PFS 4.3 months and response rate of 53% (CR, PR and SD). The authors found that previous response to anti-EGFR could predict benefit from rechallenge. Our data could not demonstrate that PR was associated with benefit from this strategy as shown in Table 2. The phase 2 study of reintroduction of cetuximab in patients previously treated with irinotecan and cetuximab-based regimens in the first line of therapy (CRICKET trial) is a recent Italian prospective trial that included 27 patients with documented disease progression during exposure to a regimen based on irinotecan and cetuximab in the first line of treatment [32]. The report ORR and DCR were 23% and 54%, respectively. These results were inferior to those of Santini *et al* [30] and are in accordance with our findings. In our study, patients submitted to rechallenge presented short median PFS of only 3.3 months as compared to previous studies and the response rate was only 19% (CR + PR). No clinical variable was related to better PFS after rechallenge in the present study. However, those patients previously treated with bevacizumab presented a non-statistically significant difference in median PFS of 6.7 months as compared to 2.9 for those that did not receive the drug.

Many advances have been achieved in the scenario of metastatic colorectal carcinoma. The notable and continuous increment in overall survival is possible by utilising active agents in the first and second line of therapy and best selection of patients based on molecular profile. Adequate care of patients leads to preserved performance status even after multiple therapies. As a result, it is becoming a very common scenario where patients have already used all available drugs and are still good candidates for treatment. To be enrolled in clinical trials would be the best option for these patients, but trials are scant in low- and middle-income countries, such as Brazil. Our cohort represents a very select group of patients with extremely high median OS. The median OS was 70 months for the 68 patients since the start of the first line and 24 months after re-treatment. It is noteworthy that 94% of our patients had a left-side primary tumour and it is known that patients with a left-sided tumour have prolonged survival when treated with anti-EGFR in the first line as demonstrated by many prospective trials [5, 6]. Therefore, re-exposure to previously used drugs becomes a strategy to be considered [15]. Anti-EGFR therapy re-exposure is a potential model to be successfully explored. Liquid biopsies over the course of the disease evolution could lead to the selection of better treatment options in the metastatic setting [33, 34]. Recently, a retrospective study of 135 patients with mCRC treated with anti-EGFR was carried out by sequencing of ctDNA with a very low allele frequency (Guardant360) platform [12]. The authors found that RAS and EGFR mutant alleles decrease exponentially over time since last anti-EGFR treatment explaining part of the benefits seen with rechallenge therapy with anti-EGFR. Moreover, the authors suggested that liquid biopsy could guide the best time of reintroduction of these agents. Anti-EGFR re-exposure is a relatively common practice in the real world. We demonstrated in this retrospective analysis that reintroduction of anti-EGFR before documented progression of disease can be beneficial for a group of patients. However, re-challenging patients that presented PD during previous treatment resulted in short PFS and few objective responses and this strategy could not be routinely advised with better patient selection with clinical and molecular tools. It should be mentioned that our patients were re-exposed after a median of four lines of therapy and most of them did not achieved partial response during first exposure. One hypothesis is that early rechallenge could have resulted in better outcomes.

## Conclusion

In summary, our data may have clinical implications since reintroduction of anti-EGFR after a break could improve quality of life without impairing the outcome for a select group of patients. Our findings must be confirmed by prospective, randomised trials designed to evaluate the reintroduction strategy and better selection of patients, and rechallenge therapy with anti-EGFR for mCRC.

## Funding

No funding was received for this study.

## Conflicts of interest

Dr Celso Mello received a congress travel grant from Merck Serono and Amgen to ASCO and ESMO meetings. The other authors have no conflicts of interest to declare.

## Ethical approval

Ethics approval for this study was approved by the local Ethic committee (CEP) at AC Camargo Cancer Center on 14/09/2018, under the number 2.894.896.

Consent to participate: NA.

Consent for publication: NA.

## Figures and Tables

**Table 1.** Patients’ characteristics.

Characteristics	% (*N*)	ReCH % (*n*)	ReIn % (*n*)
**Age (years)**			
Median/range	56.4 /29 – 85	54.2	57,16
**Gender**			
Female	39.7 (27)	47.1 (8)	37.3 (19)
Male	60.3 (41)	52.9 (9)	62.7 (32)
**Tumour location**			
Right side	4.4 (3)	0 (0)	5.9 (3)
Transverse	1.5 (1)	0 (0)	2.0 (1)
Left side	79.4 (54)	76.5 (13)	80.4 (41)
Rectum	14.7 (10)	23.5 (4)	11.8 (6
**Initial stage**			
II	5.9 (4)	11.8 (2)	3.9 (2)
III	14.7 (10)	17.6 (3)	13.7 (7)
IV	79.4 (54)	70.6 (12)	80.4 (41)
**Primary tumour resection**			
Yes	89.7 (61)	88.2 (15)	90.2 (46)
No	10.3 (7)	11.8 (2)	9.8 (5)
**Sites of metastases**			
Liver	45.6 (31)	35,3 (6)	49 (25)
Lung	11.8 (8)	17.6 (3)	9.8 (5)
Others	7.4 (5)	5.9 (1)	8 (4)
Multivisceral	35.3 (24)	41.2 (7)	33.2 (17)
**Metastasectomy (overall)**			
Yes	61.8 (42)	41.2 (7)	68.6 (35)
No	38.2 (26)	58.8 (10)	31.4 (16)
**Sites of metastasectomy**			
Liver	76.1 (32)	57.1 (4)	80.0 (28)
Lung	11.9 (5)	14.2 (1)	11.4 (4)
Peritoneum	4.7 (2)	14.2 (1)	2.8 (1)
Liver + lung + other	7.1 (3)	14.2 (1)	5.7 (2)
**Metastasectom**y **post re-exposure**			
Yes	4.4 (3)	5.8 (1)	3.9 (2)
No	95.6 (65)	94.2(16)	96.1 (48)
**RAS mutation status**			
KRAS WT	57.4 (39)	76.5 (13)	51 (26)
All RAS WT	42.6 (29)	23.5 (4)	49 (25)

**Table 2.** Treatment characteristics for entire group and ReCH and ReIn group.

Characteristics	% (*N*)	ReCH % (*n*)	ReIn % (*n*)
**Anti-EGFR—1st exposure**			
Cetuximab	94.1 (64)	100 (17)	92.2 (47)
Panitumumab	5.9 (4)	0 (0)	7.8 (4)
**Best response—1st exposure**			
SD	28.1(18)	62.5 (10)	16.7 (8)
PR	57.8 (37)	31.3 (5)	66.6 (32)
CR	12.5 (8)	0 (0)	16.7 (8)
PD	1.6 (1)	6.2 (1)	0 (0)
**Reason for 1st discontinuation**			
PD	25 (17)	100 (17)	11.8 (6)
Toxicity	8.8 (6)	0	1.0 (2)
Patient decision	1.5(1)	0	45.1(23)
Drug Holiday	33.8 (23)	0	23.5 (12)
Surgery	17.6 (12)	0	7.8 (4)
Maintenance	5.9 (4)	0	9.8 (5)
Others	7.4 (5)	0	11.8 (6)
**Line of re-exposure**			
2nd	22.1 (15)	0 (0)	29.4 (15)
3rd	39.7 (27)	29.4 (5)	43.2 (22)
≥4th	38.2 (26)	70.6 (12)	27.4 (14)
